# Knowledge, attitudes, and practices regarding antimicrobial use and resistance among smallholder dairy farmers in Zambia: a cross-sectional study

**DOI:** 10.3389/fvets.2026.1731209

**Published:** 2026-04-23

**Authors:** Inyambo Mumbula, Steward Mudenda, Lydia Trupe, Lysa Muila Thendji, Anil Gorle, Aisling Glennie, Claire Gilbert, Alison Pyatt, John Bwalya Muma, Doreen Sitali

**Affiliations:** 1 Department of Disease Control, School of Veterinary Medicine, University of Zambia, Lusaka, Zambia; 2 Department of Health Promotion and Education, School of Public Health, University of Zambia, Lusaka, Zambia; 3 Department of Pharmacy, School of Health Sciences, University of Zambia, Lusaka, Zambia; 4 Veterinary Medicines Directorate of the United Kingdom, Addlestone, United Kingdom

**Keywords:** antimicrobial use, antimicrobial resistance, dairy farming, knowledge attitude and practices, smallholder farmers, Zambia

## Abstract

**Introduction:**

The irrational use of antibiotics in livestock encourages the development of antimicrobial resistance (AMR), which poses a risk for human and animal health. This study assessed knowledge, attitude and practices (KAP) regarding antimicrobial use (AMU) and AMR among smallholder dairy farmers in Zambia.

**Methods:**

A cross-sectional survey was conducted among 617 farmers selected using a cluster-randomized proportionate sampling strategy across six districts between August and October 2024. KAP scores were computed based on 16 knowledge, 12 attitudes, and 14 practice items. Correct or appropriate responses were scored as “1” and incorrect or inappropriate responses as “0”. For knowledge, “not sure” responses were coded as incorrect, and for attitudes and practices, “sometimes” responses were coded as inappropriate to prioritize adherence to stewardship standards. Domain scores were summed and converted to percentages. For comparability to previous studies in similar regional contexts, cut-offs of 60% for knowledge, 58% for attitude and 65% for practice were adopted to classify levels as appropriate or positive rather than as validated standards. Statistical associations were identified using multivariate logistic regression adjusted with cluster-robust standard errors.

**Results:**

Of the 617 participants, the majority displayed inappropriate knowledge (70.99%) and inappropriate practices (59.97%), though 64.34% held positive attitudes. Key factors associated with higher odds of appropriate knowledge included milking dairy cattle only (AOR = 4.34, 95% CI: 1.956–9.651, *p* < 0.001) and membership in a cattle cooperative (AOR = 3.35). Farmers with tertiary education (AOR = 15.04, 95% CI: 3.39–66.77, *p* < 0.001) and those who listened to cattle management radio programs (AOR = 2.02, 95% CI: 1.24–3.38, *p* < 0.001) also demonstrated significantly better knowledge. However, longer farming experience (over 11 years) was associated with lower odds of appropriate practices compared to those with less than 5 years of experience (AOR = 0.49, 95% CI: 0.31–0.79, *p* = 0.003).

**Conclusion:**

These KAP levels among smallholder dairy farmers are consistent with conditions that facilitate antimicrobial misuse and the potential spread of AMR. While cooperatives and radio programs are associated with knowledge awareness, coupling them with interventions addressing structural barriers and enforcing prescription-only regulations may bridge the gap between knowledge and practice.

## 1 Introduction

Antimicrobial resistance (AMR) has emerged to become a global health problem of pandemic proportions (1). Though a natural process, AMR is exacerbated by irrational use and misuse of antimicrobials (2). Livestock production is the largest global consumer of antimicrobials and represents an important risk factor for the development of AMR (3). In a study by Ardakani et al. (4) cattle production accounted for the largest global antimicrobial use representing 53.5% of total antimicrobial use (AMU) among chicken, pigs and cattle. In dairy cattle production, usage of antimicrobials is common for diseases such as mastitis which threaten cattle health and milk productivity (5). However, the unnecessary use of antibiotics in cattle treatment, coupled with poor antimicrobial stewardship (AMS) practices such as failure to observe withdrawal periods have been shown to be associated with the emergence and spread of AMR (6). In addition, antimicrobial residues in milk can pose risks of transmission to humans through consumption, exacerbating the development of resistant bacterial strains in humans (7).

It is anticipated that AMR will be the leading cause of death by the year 2050 in the absence of present interventions (8). The interventions should take into consideration not only the input from medical sciences but also the input from social, agricultural and environmental sciences (9). This multidisciplinary approach is a recognition of the “one health” nature of AMR transcending beyond the human health sector to include plant, animal health and environmental sectors (10). Interventions should focus on enhancing knowledge and attitudes regarding AMU and AMR, improving antimicrobial use (AMU) practices, and surveillance of AMR, to contribute to the reduction in AMR development and spread (7, 11).

In Zambia, the dairy sector is mainly led by smallholder farmers which includes traditional smallholder livestock farmers and emerging formal small-scale dairy farms accounting for more than 80% of the total milk produced (12). Smallholder dairy farming is often a family-owned enterprise on an average land size of 10 hectares characterized by extensive local breeds production for beef among traditional producers and semi-intensive production with mixed-breed cows in semi-intensive production systems (13, 14). Smallholder dairy farming creates jobs, meets nutritional needs, and is a source of income for both food and non-food expenses thereby reducing poverty and food insecurity within rural Zambia (15). However, maximum profit potential is not realized due to low yields of milked cattle breeds and poor production practices such as infection prevention and control and disease management which reduce productivity and make volumes less enticing for processors (13, 16). As such, most of the milk produced by smallholder dairy farmers is traded informally (13). To address this, the initiative of dairy cooperatives pools together the affiliated farmers' milk volumes, regulates milk quality, cushions single farmer marketing burdens and commands stronger bargaining power within the formal sector through milk collection centers (MCCs) (13, 16).

Mastitis is one of the major reasons for which milk is rejected at MCCs Zambia, leading to reliance on antibiotics for disease management (16). Drugs such as ampicillin, sulfamethoxazole, cefpodoxime, and gentamicin have been reported to be resistant to *E. coli* in Zambia (17), contributing factors to this resistance include disease prevalence (e.g., lumpy skin), farm size, cattle breed, geographical location and general management practices such as biosecurity and hygiene practices (17). Even though data on the knowledge, attitudes and practices regarding AMU and AMR among dairy cattle farmers remains scarce, evidence from other sub-Saharan African countries shows that farmers' KAP regarding AMU is influenced by the education level and years of experience (2, 18–21). In Ethiopia and Kenya for instance, studies report that a significant proportion of dairy farmers used antibiotics without veterinary prescriptions (19, 21). Furthermore, this “knowledge action gap” has been observed in Uganda, where AMR beliefs were only weakly associated with actual prudent AMU (20). In the same study, awareness of AMR was not associated with desirable practices such as adhering to withdrawal periods (20). Building on this regional evidence, it is important to understand how localized socio-demographic factors including cooperative membership and access to cattle management radio programs influence KAP on AMR and AMU. This study therefore evaluates the KAP regarding AMR and AMU and the associated socio-demographic factors among smallholder dairy farmers in Zambia.

## 2 Materials and methods

### 2.1 Study design and setting

**Figure 1:**
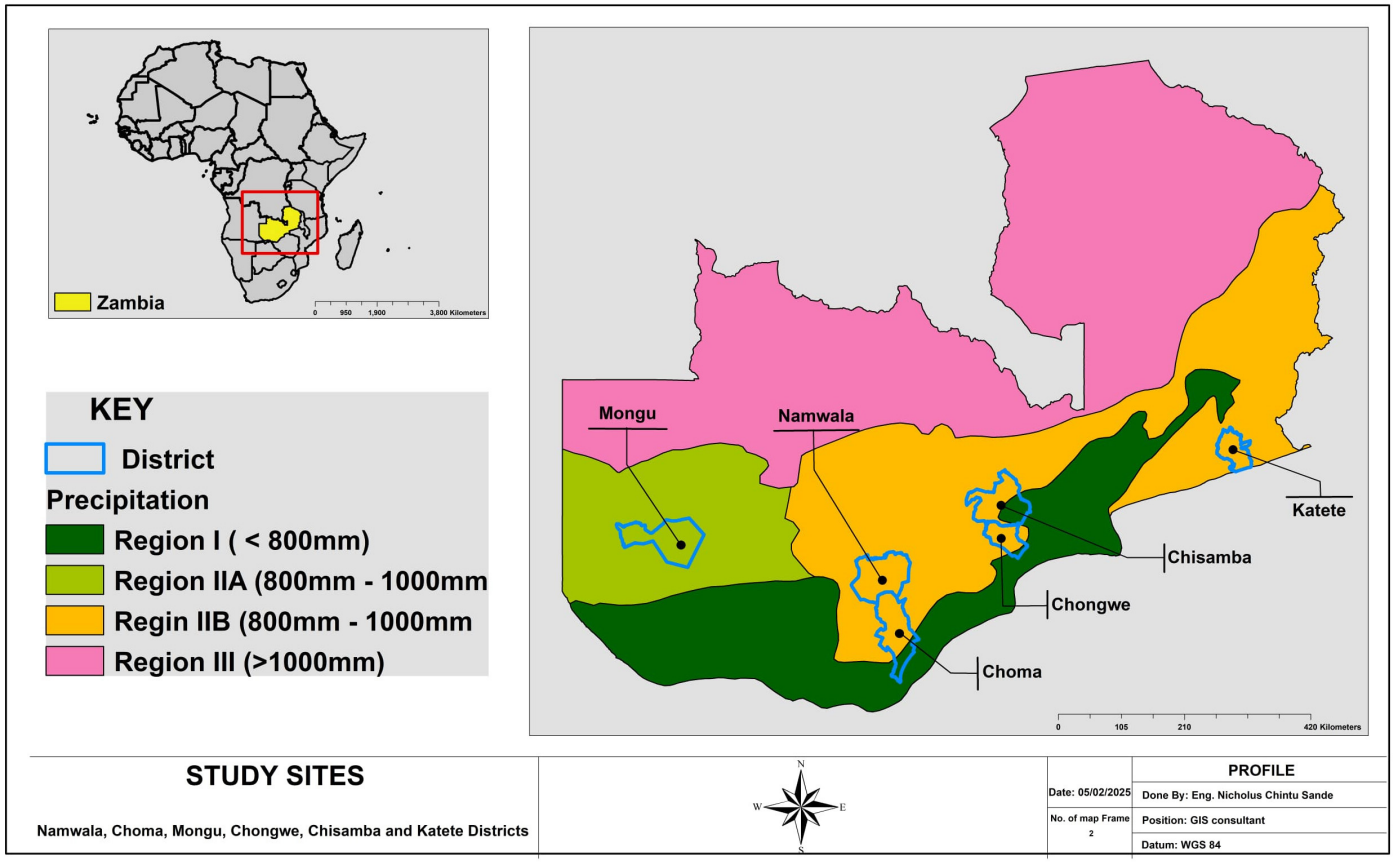
Map of Zambia showing the districts in which the study was conducted.

A cross-sectional study design was used to assess small holder dairy farmers' KAP regarding AMU and AMR from August to October 2024. The study was conducted in six districts that is Chongwe (Lusaka Province), Chisamba (Central Province), Katete (Eastern Province), Namwala (Southern Province), Choma (Southern Province) and Mongu (Western Province) across five provinces of Zambia (Figure 1), all located within agroecological region II, which hosts the largest cattle population in the country (22). The districts were selected to represent major dairy production areas within this high cattle-density belt.

### 2.2 Study population and sample size

The study population consisted of smallholder dairy farmers aged 18 years and older from the selected districts. For the purpose of this study, smallholder dairy farmers were defined as cattle farmers operating limited-resource production systems, relying primarily on family labor, and managing relatively small cattle herds (fewer than 20 exclusively dairy cattle animals) or traditionally farming cattle primarily for beef with milk production directed toward household consumption and/or local market sales (16). Exclusion criteria included non-dairy cattle farmers and questionnaires with substantial missing data on key KAP variables. A total of 21 responses were excluded from analysis due to ineligibility or incomplete data.

The sample size for this study was calculated using the Cochrane's formula for sample size calculation designed for determining a representative sample size for proportions.


$$\begin{array}{l} n=\frac{Z^{2}p\left(1-p\right)}{e^{2}} \end{array}$$ (1)

Where: Z = 1.96 at 95% confidence level, *p* = 0.47 proportion of AMR awareness reported by Hirwa et al. (23), and *e* = 0.05 margin of error. This yielded an initial sample size of 383 participants. A design effect of 1.5 was applied to account for clustering at the district level, resulting in 575 participants. After adjusting for a 10% non-response rate, the final sample size was 638 participants.

### 2.3 Data collection and sampling strategy

A cluster randomized proportionate sampling strategy was used, with districts serving as clusters. Six districts were included in the study, and the sample size was allocated proportionally based on the estimated number of smallholder dairy farmers in each district. Within each district, farmers were randomly selected from cooperative and milk collection center lists using a simple random sampling approach. Data collection was done using Kobo Collect (v2024.2.4) mobile app installed on Android phones and uploaded to the Kobo toolbox server for storage (https://support.kobotoolbox.org/kobocollect_on_android_latest.html). The questionnaire was designed to address the study objectives and comprised 54 questions across four sections: 12 items on demographic information of participants, 16 items assessing knowledge of antibiotics, antibiotic use and AMR, 12 items exploring attitudes toward antibiotic use and AMR, and 14 items examining practices related to antibiotic use and disease control. Boolean and 3-point Likert scale questions were used for the KAP sections of the questionnaire. Internal consistency was assessed separately for each KAP subscale. Cronbach's alpha was 0.92 for the knowledge scale, 0.76 for the attitude scale, and 0.73 for the practice scale, indicating excellent internal consistency for knowledge and acceptable reliability for both the attitude and practice subscales.

### 2.4 Ethical considerations

Ethical approval was obtained from the University of Zambia Biomedical Research Ethics Committee (REF. No. No. 5523-2024), and the National Health Research Authority (NHRA-1416/26/07/2024) before data collection commenced. Written informed consent to participate in the study was obtained from each participant. All data presented in this report has been deidentified to honor participant anonymity. Data were stored in password-protected computer folders and were accessible only to authorized members of the research team.

### 2.5 Data analysis and management

An Excel spreadsheet with Microsoft 365 was downloaded for coding. Coded data were imported into STATA version 15 for cleaning and subsequent statistical analysis. Data cleaning involved deletion of missing values and responses from ineligible respondents. The entries did not have any duplicates. Of the 638 responses collected, 13 (2.0%) contained missing data on key KAP items required for score construction and were excluded from analysis. A complete-case approach was applied, as the proportion of missing data was low and limited to key variables necessary for KAP score calculation. Additionally, four respondents were excluded because they were not engaged in dairy production, and four were excluded for being younger than 18 years. After these exclusions, the final sample consisted of 617 participants. As the sample size calculation incorporated a 10% non-response adjustment and the final sample exceeded the minimum design-effect-adjusted requirement (*n* = 575), statistical power was maintained.

Descriptive statistical analysis was used to summarize demographics, knowledge, attitudes and practice related items as frequencies and percentages. To determine the KAP levels, correct responses were scored as “1” and the incorrect responses were scored as “0.” For knowledge items, “not sure” responses were coded as incorrect, consistent with standard KAP methodology where uncertainty reflects lack of accurate knowledge. For practice items, responses such as “sometimes” were coded as inappropriate practice when full adherence was required according to antimicrobial stewardship recommendations. While this approach simplifies the behaviors into binary categories, it facilitates comparability with previous KAP studies and allows clear classification of appropriate vs. inappropriate practices (23–26).

In the absence of a validated external gold standard for defining appropriate knowledge, attitudes, and practices regarding antimicrobial use and resistance in this context, a cut-off of 60% knowledge, 58% attitude, and 65% practice determined “good” and “poor” KAP levels adopted from a previously KAP study conducted in a similar setting (27). This approach was selected to ensure methodological consistency and facilitate comparability of findings within similar regional contexts. As no validated external gold standard or independent outcome measure existed for defining appropriate KAP levels in this context, ROC analysis was not methodologically applicable. Knowledge and practice domains were categorized as “appropriate” or “inappropriate” based on adherence to recommended antimicrobial use principles. Attitude scores were categorized as “positive” or “negative” to reflect favorable or unfavorable perceptions toward antimicrobial stewardship. Inferential statistics included a Chi-square test of association between demographic characteristics and KAP levels. Demographic variables that had a *p*-value less than 0.20 during the bivariable analysis were included in a multivariable logistic regression analysis as recommended by Malhotra (28), to determine the key factors that affect KAP of smallholder dairy farmers toward AMU and AMR in Zambia. Multivariable model development was investigator-led and guided by epidemiological relevance and theoretical considerations. Variables were removed sequentially while assessing their influence on confounding, model stability, and overall fit. Spearman's rank correlation coefficients were computed to assess associations between knowledge, attitude, and practice scores, as normality assumptions were not met for two of the three domain scores. To account for the clustered sampling design (district-level clustering) in the multivariable logistic regression, analysis was adjusted using cluster-robust standard errors in STATA in order to account for intra-cluster correlation and to provide reliable variance estimates. All statistical analyses were considered significant at 95% confidence level and *p*-value <0.05.

### 2.6 Model diagnostics

For model diagnostics, each fitted multivariable logistic regression model, multicollinearity among predictors was assessed using variance inflation factors (VIF), with VIF values below 5 considered indicative of acceptable collinearity. All VIF values were below 5, indicating no evidence of problematic collinearity. Model specification was guided by theoretical relevance and bivariable screening (*p* < 0.20), and final models were selected based on parsimony and goodness-of-fit criteria. Competing models were compared using Akaike Information Criterion and Bayesian Information Criterion, with lower values indicating improved model fit. Likelihood ratio tests were performed to assess overall model significance. Model performance and discrimination were evaluated using the area under the Receiver Operating Characteristic curve which demonstrated acceptable to excellent discriminatory ability across models.

## 3 Results

### 3.1 Socio-demographic characteristics

A total of 617 participants were included in the analysis with most participants being males (86.55%) and married (83.79%). The median age of the participants was 42 years (IQR = 32, 53) with the majority (*n* = 214) aged between 31 and 45 years. Only 8% of the participants milked exotic dairy breeds (Friesian or Jersey). Most participants reported selling milk to milk collection centers (44.89%), and more than half (56.24%) were members of a cattle cooperative. Most participants were farm owners (75.85%) with the majority (*n* = 176) having between 11 and 20 years of experience. The median number of years in cattle rearing was 12 years (IQR: 6, 21). Finally, 67.10% of participants reported listening to radio programs related to cattle management (see Table 1).

**Table 1:** Socio- demographic characteristics of dairy cattle farmers.

Variable	Frequency	Percentage (%)
Age		
18–30	143	23.18
31–45	214	34.68
46–60	179	29.01
>61	81	13.13
Gender		
Female	83	13.45
Male	534	86.55
Marital status		
Single	100	16.21
Married	517	83.79
Education level		
None	34	5.51
Primary	271	43.92
Secondary	278	45.06
Tertiary	34	5.51
Other occupation		
No	519	84.12
Yes	98	15.88
Type of cattle milked		
Beef	284	46.03
Mixture	281	45.54
Dairy (Friesian or Jersey)	52	8.43
Where milk is sold		
Do not sell	157	25.45
Sell in to MCC	277	44.89
Sell in community	164	26.58
Sell in both MCC & community	19	3.08
Years of experience rearing cattle		
0–5 years	139	22.53
6–10 years	146	23.66
11–20 years	176	28.53
>21 years	156	25.28
Position at farm		
Owner	468	75.85
Farm manager	30	4.86
Caretaker	68	11.02
Other	51	8.27
Belong to cooperative		
No	270	43.76
Yes	347	56.24
Listens to radio or TV on cattle management		
No	203	32.90
Yes	414	67.10
	median	IQR
Age (in years)	42	32–53
Years of experience	12	6–21′

### 3.2 Characteristics of KAP composite scores

The knowledge scale comprised 16 binary-scored items (0 = incorrect, 1 = correct), yielding a theoretical range of 0–16. Observed scores ranged from 0 to 15, with a mean of 5.3 (SD = 4.7). Internal consistency was excellent (Cronbach's α = 0.92). The attitude scale consisted of 12 items (range 0–12). Observed scores ranged from 0 to 12, with a mean of 7.4 (SD = 2.7). Internal consistency was acceptable (α = 0.76). The practice scale included 14 items (range 0–14). Observed scores ranged from 3 to 12, with a mean of 7.9 (SD = 1.9). Internal consistency was acceptable (α = 0.73).

### 3.3 Knowledge about AMR and AMU

**Figure 2:**
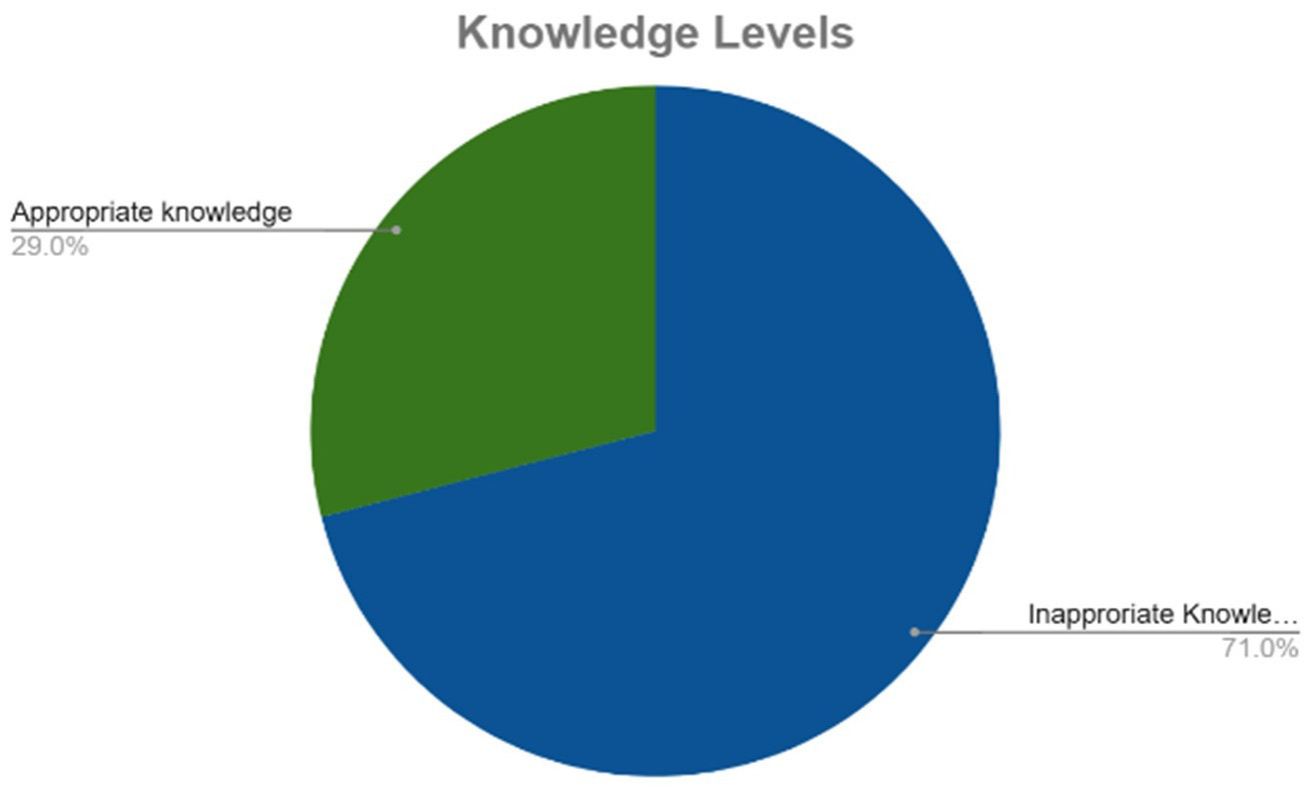
Knowledge levels.

A summary of participants' results regarding their general knowledge of AMU and AMR are included in Table 2. Over half (55.27%) of participants had heard about the word “antibiotics” even though only 41.82% were able to correctly distinguish antibiotics from other antimicrobials. Majority (67.10%) of participants thought that antibiotics could be used to treat all cattle diseases and over 80.23% of participants felt that antibiotics were used for disease prevention. Only 30.15% of participants could define antibiotic resistance correctly and 17.99% of participants thought that using expired antibiotics could cause antibiotic resistance. Overall, 70.99% of participants had inappropriate knowledge regarding antimicrobial use and resistance as shown in Figure 2.

**Table 2:** Knowledge of participants on AMU and AMR (*N* = 617).

AMU/AMR knowledge related items	Yes *n* (%)	No *n* (%)
Have you ever heard of the term “antibiotics”?	**341 (55.27)**	276 (44.73)
Antibiotics are medicines that kill bacteria	**203 (32.90)**	414 (67.10)
Able to give a correct example of an antibiotic?	**258 (41.82)**	359 (58.18)
Do antibiotics kill viruses	330 (53.48)	**287 (46.52)**
Should antibiotics be used for growth promotion?	599 (97.08)	**18 (2.92)**
Should antibiotics be used for preventing diseases?	495 (80.23)	**122 (19.77)**
Should antibiotics be used for treating all cattle diseases	324 (52.51)	**293 (47.49)**
Should antibiotics be used to increase milk production?	613 (99.35)	**4 (0.65)**
Should antibiotics be used to treat bacterial cattle diseases only?	**203 (32.90)**	414 (67.10)
Have you ever heard of the term antibiotic resistance?	**214 (34.68)**	403 (65.32)
Antibiotic resistance is the failure of antibiotics to destroy bacteria	**186 (30.15)**	431 (69.85)
Overuse of antibiotics can cause antibiotic resistance	**180 (29.17)**	437 (70.83)
Use of expired antibiotics can contribute to antibiotic resistance	**111 (17.99)**	506 (82.01)
Obtaining veterinary antibiotic prescription prevents antibiotic resistance	**177 (28.69)**	440 (71.31)
Using the correct dose of antibiotics to treat cattle prevents antibiotic resistance	**211 (34.20)**	406 (65.80)
The withdraw period allows for antibiotics to wear off in cattle and avoid antibiotic residues in meat or milk	**448 (72.61)**	169 (27.39)
Overall respondents' knowledge score (cutoff = 60%)	Appropriate	Inappropriate
	**179 (29.01)**	438 (70.99)

Responses in bold indicate appropriate knowledge.

### 3.4 Attitudes about AMR and AMU

**Figure 3:**
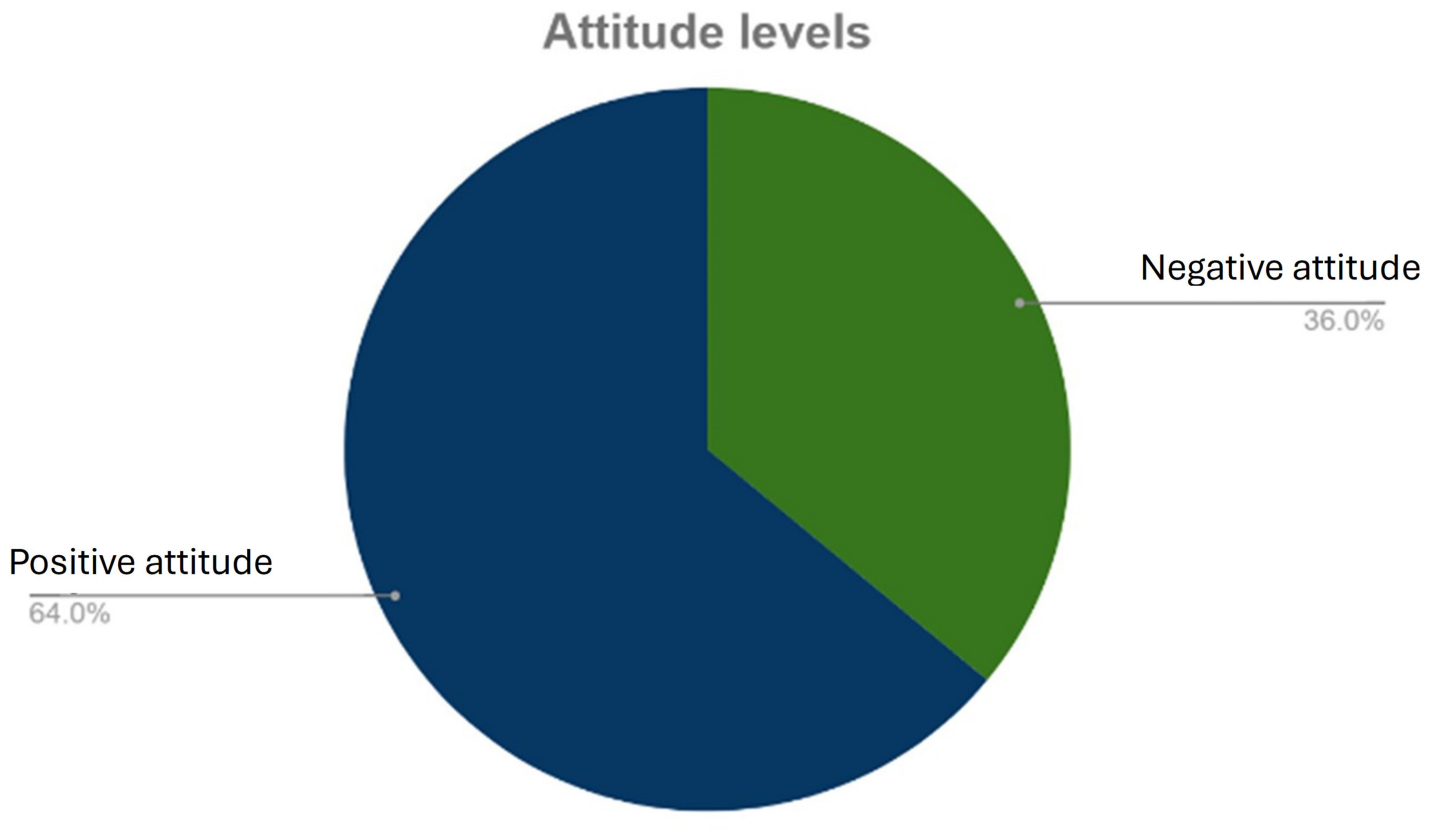
Attitude levels.

Regarding participants' attitudes, Table 3 shows that a majority of respondents (66.45%) agreed that antibiotic treatment could be stopped once symptoms improve, indicating a negative attitude toward completing the full course of antibiotics. However, the majority (88.01%) preferred keeping antibiotics at home for later use. Still a majority (97.24%) of participants felt that checking the expiry date of antibiotics was important to avoid antibiotic resistance. 52.67% of participants preferred obtaining antibiotics from colleagues even though many (95.62%) felt that it was important to follow a veterinarian's prescription to treat their cattle. Regardless, 52.51% of participants felt it was acceptable to buy antibiotics without a prescription. An overall positive attitude (64.34%) toward AMU and AMR was found among participants as shown in Figure 3.

**Table 3:** Participants' attitude toward AMU and AMR (*N* = 617).

AMU/AMR attitude related items	Agree *n* (%)	Disagree *n* (%)
I would stop the full course once symptoms improve	410 (66.45)	**207 (33.55)**
Misusing antibiotics can lead to antibiotic resistance	**382 (61.91)**	235 (38.09)
I find nothing wrong with buying antibiotics without a prescription	293 (47.49)	**324 (52.51)**
I prefer keeping antibiotics at home for later use	543 (88.01)	**74 (11.99)**
Checking the expiry date of antibiotics is important to avoid antibiotic resistance	**600 (97.24)**	17 (2.76)
I would follow a veterinarian's prescription to treat my animals	**590 (95.62)**	27 (4.38)
I would use antibiotics for disease prevention	251 (40.68)	**366 (59.32)**
I prefer obtaining antibiotics from colleagues	325 (52.67)	**292 (47.33)**
I can use the same antibiotic for all cattle diseases	197 (31.93)	**420 (68.07)**
Antibiotic resistance does not have any effects on human health	301 (48.78)	**316 (51.22)**
Antibiotic resistance does not have any effect on cattle health	204 (33.06)	**413 (66.94)**
Antibiotic resistance is not a farmer's concern	183 (29.66)	**434 (70.34)**
Overall respondents' attitude score (Cutoff = 58%)	Positive	Negative
	**397 (64.34)**	220 (35.66)

Responses in bold indicate a positive attitude.

### 3.5 Practices related to AMU and AMR

**Figure 4:**
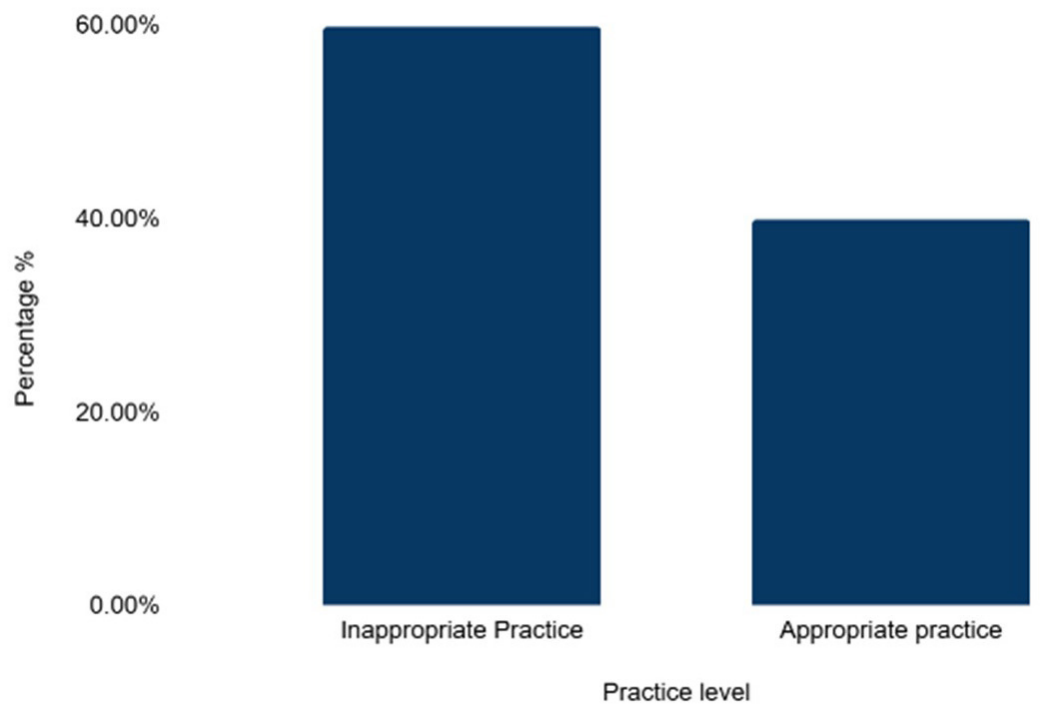
Practice levels.

Even though most (94.81) of participants used antibiotics to treat their animals, a significant proportion also used them for disease prevention (59.16%) and pain relief (66.29%). A significant proportion (60.20%) of participants reported that they always consulted a veterinarian before treating their cattle, while 72.61% asked the veterinarian to prescribe their preferred antibiotic. About 76.50% checked the required dose before administering antibiotics, and 96.27% always checked the expiry date of antibiotics before administering them. Even though milk withdrawal period was observed by close to 93% of the participants, only 24% participants adhered to the withdraw periods for the consumption of meat. The majority (59.97%) had an overall inappropriate practice score regarding antimicrobial use and resistance as shown in Table 4 below and as shown in Figure 4.

**Table 4:** Practices of dairy farmers on AMU and AMR (*N* = 617).

AMU/AMR practice related items	Yes *n* (%)	No *n* (%)
Do you use antibiotics for treatment of bacterial diseases	**585 (94.81)**	32 (5.19)
Do you use antibiotics to prevent bacterial cattle disease?	365 (59.16)	**252 (40.84)**
Do you use antibiotics for cattle pain management?	409 (66.29)	**208 (33.71)**
Do you buy antibiotics from the agrovet store after obtaining a prescription from a vet staff?	**326 (52.84)**	291 (47.16)
Do you always consult a veterinarian before treating your cattle?	**372 (60.29)**	245 (39.71)
Do you ask for your own preferred antibiotics from a veterinarian	448 (72.61)	**169 (27.39)**
Do you consult a veterinarian when antibiotic course finishes, but cattle is not cured?	**391 (63.37)**	226 (36.63)
Do you check the required dose before treating your cattle?	**472 (76.50)**	145 (23.50)
Do you check the expiry date before administering your antibiotics?	**594 (96.27)**	23 (3.73)
Do you believe other farmers' recommendations for treating your animals?	446 (72.29)	**171 (27.71)**
Do you share antibiotics with other farmers?	469 (76.01)	**148 (23.99)**
Do you observe the withdrawal period for milk?	**573 (92.87)**	44 (7.13)
Do you observe the withdrawal period for meat consumption?	**149 (24.15)**	468 (75.85)
Do you treat healthy cattle with antibiotics to avoid waste?	159 (25.77)	**458 (74.23)**
Overall respondents' practices score (cutoff = 65%)	Appropriate	Inappropriate
	**247 (40.03)**	370 (59.97)

Responses with in bold indicate appropriate practices.

### 3.6 Associations of socio-demographic characteristics with dairy farmers' KAP

Significant associations between respondents' knowledge levels and socio-demographic factors were recorded (Table 5). Knowledge levels were significantly associated with age (*p* = 0.003), education level, type of cattle milked, belonging to a cattle cooperative (*p* < 0.001) and listening to cattle management radio programs (*p* = 0.025). Attitude levels were significantly associated with age (*p* = 0.025), education level, having other occupation (*p* = 0.001), type of cattle milked (*p* = 0.01), where milk was sold (*p* = 0.008), belonging to a cattle cooperative and listening to cattle management radio programs (*p* < 0.001). Appropriate practices were significantly associated with age (*p* = 0.015), type of cattle milked (*p* < 0.001), years of experience (*p* = 0.001), education levels and position held on farm (*p* = 0.002). Belonging to a cooperative or listening to cattle management programs were not associated with appropriate practices, *p* values = 0.399 and 0.148 respectively.

**Table 5:** Associations of socio- demographic characteristics with knowledge, attitudes, and practices.

Variable	Knowledge		Attitude		Practice	
	Appropriate *f* (%)	*p*-value	Positive *f* (%)	*p-*value	Appropriate *f* (%)	*p*-value
Age (in years)						
18–30	30 (20.98)	0.003	79 (55.24)	0.025	71 (49.65)	0.015
31–45	56 (26.17)		144 (67.29)		73 (34.11)	
46–60	58 (32.40)		114 (63.69)		66 (36.87)	
>61	35 (43.21)		60 (74.07)		37 (45.68)	
Gender						
Male	163 (30.52)	0.036	353 (66.10)	0.021	216 (40.03)	0.592
Female	16 (19.28)		44 (53.01)		31 (37.35)	
Marital status						
Single	19 (19.00)	0.016	61 (61.00)	0.446	44 (44.00)	0.376
Married	160 (30.95)		336 (64.99)		203 (39.26)	
Education level						
None	3 (8.82)	<0.001	23 (67.65)	0.001	22 (64.71)	0.002
Primary	55 (20.30)		159 (58.67)		100 (36.90)	
Secondary	99 (35.61)		184 (66.19)		105 (37.77)	
Tertiary	22 (64.71)		31 (91.18)		20 (58.82)	
Other occupation						
No	144 (27.75)	0.111	320 (61.66)	0.001	204 (39.31)	0.397
Yes	35 (35.71)		77 (78.57)		43 (43.88)	
Type of cattle milked						
Beef cattle only	67 (23.59)	<0.001	107 (37.68)	0.010	209 (73.59)	<0.001
Both dairy and beef	85 (30.25)		109 (38.79)		153 (54.45)	
Dairy cattle only	27 (51.92)		31 (59.62)		35 (67.31)	
Where milk is sold						
Do not sell	29 (18.47)	<0.001	86 (54.78)	0.007	67 (42.68)	0.603
Sell to MCC	85 (28.72)		206 (69.59)		119 (40.20)	
Sell to community	65 (39.63)		105 (64.02)		61 (37.20)	
Years of experience						
0–5 years	37 (26.62)	0.336	85 (61.15)	0.281	75 (53.96)	0.001
6–10 years	36 (24.66)		88 (60.27)		60 (41.10)	
11–20 years	54 (30.68)		115 (65.34)		61 (34.66)	
>21 years	52 (33.33)		109 (69.87)		51 (32.69)	
District						
Namwala	8 (7.27)	<0.001	70 (63.64)	0.018	22 (20.00)	<0.001
Choma	26 (32.10)		63 (77.78)		31 (38.27)	
Mongu	49 (42.98)		70 (61.40)		31 (27.19)	
Chongwe	38 (42.70)		64 (71.91)		60 (67.42)	
Chisamba	31 (26.72)		64 (55.17)		45 (38.79)	
Katete	27 (25.23)		66 (61.68)		58 (54.21)	
Position at farm						
Owner	136 (29.06)	0.003	304 (64.96)	0.173	176 (37.61)	0.002
Farm manager	15 (50.00)		23 (76.67)		20 (66.67)	
Caretaker	22 (32.35)		43 (63.24)		35 (51.47)	
Other	6 (11.76)		27 (52.94)		16 (31.37)	
Belonging to a cattle cooperative						
No	43 (15.93)	<0.001	152 (56.30)	<0.001	103 (38.15)	0.399
Yes	136 (39.19)		245 (70.61)		144 (41.50)	
Listen to radio on cattle management						
No	47 (23.15)	0.025	100 (49.26)	<0.001	73 (35.96)	0.148
Yes	132 (31.88)		297 (71.74)		174 (42.03)	

### 3.7 Correlation between the KAP scores

**Figure 5:**
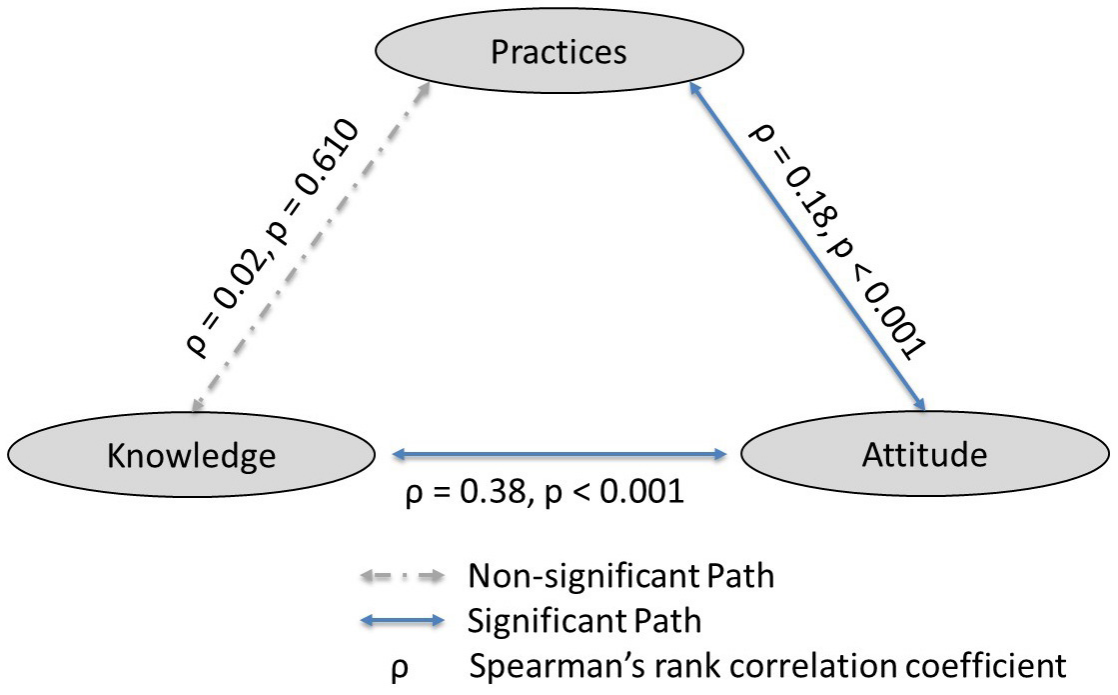
Spearman's rank KAP correlations.

Spearman's rank correlation analysis was performed to assess relationships among the KAP scores. Knowledge and attitude scores demonstrated a significant positive correlation (ρ = 0.38, *p* < 0.001), indicating a moderate association. Attitude and practice scores showed a weak but statistically significant positive correlation (ρ = 0.18, *p* < 0.001). In contrast, the correlation between knowledge and practice scores was negligible and not statistically significant (ρ = 0.02, *p* = 0.610) as Figure 5 shows.

### 3.8 Logistic regression analyses for KAP

Farmers that milked dairy cattle only were 4 times more likely to have appropriate knowledge than those milking beef cattle only (AOR = 4.34, 95% CI: 1.956–9.651, *p* < 0.001). Farmers older aged between 46 and 60 years had about twice higher odds of appropriate knowledge compared to those younger than 30 years, with farmers aged 46–60 years having an AOR of 1.97 (95% CI: 1.052–3.683) and those above 61 years having an AOR of 2.46 (95% CI: 1.12–5.06, *p* = 0.014). Farmers who reported listening to radio programs on cattle management had higher odds of appropriate knowledge compared with those who did not (AOR = 2.02, 95% CI: 1.24–3.38, *p* < 0.001). Farmers with secondary education had higher odds of appropriate knowledge compared with those without formal education (AOR = 5.88, 95% CI: 1.67–20.64, *p* = 0.006). Those with tertiary education also showed substantially higher odds of appropriate knowledge (AOR = 15.04, 95% CI: 3.39–66.77, *p* < 0.001), although the wide confidence interval may suggest reduced precision. Members of cooperatives were three times more likely to report appropriate knowledge than those who weren't (AOR = 3.35, 95% CI: 2.162–5.199, *p* < 0.01) (Table 6).

**Table 6:** Logistic regression analyses of the independent factors associated with Knowledge on AMU and AMR.

Predictor variables	Knowledge					
	Unadjusted			Adjusted		
	Odds ratio	*p*-value	Confidence interval	Odds ratio	*p*-value	Confidence interval
Age (in years)						
18–30	1 (*ref*)			1 *(ref)*		
31–45	1.335	0.262	0.805–2.213	1.333	0.351	0.722–2.469
46–60	1.806	0.023	1.084–3.008	1.968	0.031	1.052–3.683
>61	2.866	0.001	1.578–5.204	2.462	0.014	1.197–5.06
Gender						
Female	1 *(ref)*			1 *(ref)*		
Male	1.839	0.038	1.034–3.273	2.769	0.005	1.289–5.945
Marital status						
Single	1 *(ref)*			1 *(ref)*		
Married	1.911	0.017	1.120–3.258	1.673	0.124	0.881–3.177
Education level						
None	1 *(ref)*			1 *(ref)*		
Primary	2.631	0.121	0.774–8.934	2.715	0.125	0.771–9.566
Secondary	5.715	0.005	1.702–19.189	5.876	0.006	1.672–20.644
Tertiary	18.944	<0.001	4.770–75.242	15.043	<0.001	3.390–66.765
Type of cattle milked						
Beef cattle only	1 *(ref)*			1 *(ref)*		
Both dairy and beef	1.404	0.075	0.966–2.042	1.612	0.034	1.036–2.510
Dairy cattle only	3.498	<0.001	1.901–6.434	4.344	<0.001	1.956–9.651
Where milk is sold						
Do not sell	1 *(ref)*			1 *(ref)*		
Sell to MCC	1.778	0.018	1.105–2.861	0.689	0.201	0.389–1.220
Sell to community	2.898	<0.001	1.739–4.829	2.233	0.006	1.256–3.970
Belonging to a cattle cooperative						
No	1 *(ref)*			1 *(ref)*		
Yes	3.403	<0.001	2.301–5.031	3.353	<0.001	2.162–5.199
Listen to radio on cattle management						
No	1 *(ref)*			1 *(ref)*		
Yes	1.554	0.025	1.056–2.286	2.018	0.003	1.242–3.379

Hosmer-Lemeshow goodness-of-fit test statistic for knowledge: 5.99, *p* = 0.6488; ROC curve – 0.7886.

Regarding attitudes, farmers aged 31–45 years (AOR = 1.81, 95% CI: 1.10–2.97, *p* = 0.014) and those older than 61 years (AOR = 2.35, 95% CI: 1.21–4.56, *p* = 0.011) had higher odds of positive attitudes compared with farmers aged 18–30 years. Male farmers were also more likely to exhibit positive attitudes than females (AOR = 1.80, 95% CI: 1.05–3.08, *p* = 0.031). Farmers with an additional occupation had higher odds of positive attitudes compared with those without (AOR = 1.95, 95% CI: 1.15–3.33, *p* = 0.013). Membership in a cattle cooperative was significantly associated with positive attitudes (AOR = 2.05, 95% CI: 1.41–2.97, *p* < 0.001), as was listening to radio programs on cattle management (AOR = 2.28, 95% CI: 1.56–3.33, *p* < 0.001). In contrast, farmers who kept both dairy and beef cattle had lower odds of positive attitudes compared with those keeping beef cattle only (AOR = 0.45, 95% CI: 0.31–0.66, *p* < 0.001) (see Table 7).

**Table 7:** Logistic regression analyses of the independent factors associated with attitudes toward AMU and AMR.

Predictor variables	Attitude					
	Unadjusted			Adjusted		
	Odds ratio	*p*-value	Confidence interval	Odds ratio	*p*-value	Confidence interval
Age (in years)						
18–30	1 *(ref)*			1 *(ref)*		
31–45	1.666	0.022	1.077–2.578	1.812	0.014	1.104–2.972
46–60	1.421	0.125	0.907–2.226	1.479	0.121	0.901–2.426
>61	2.315	0.006	1.275–4.202	2.351	0.011	1.214–4.557
Gender						
Female	1 *(ref)*			1 *(ref)*		
Male	1.729	0.022	1.083–2.758	1.801	0.031	1.054–3.077
Education level						
None	1 *(ref)*			1 *(ref)*		
Primary	0.679	0.317	0.317–1.449	0.625	0.254	0.280–1.400
Secondary	0.936	0.865	0.437–2.003	0.780	0.552	0.344–1.767
Tertiary	4.942	0.024	1.235–19.782	3.894	0.071	0.906–16.742
Other occupation						
No	1 *(ref)*			1 *(ref)*		
Yes	2.280	0.002	1.363–3.814	1.954	0.013	1.149–3.325
Type of cattle milked						
Beef cattle only	1 *(ref)*			1 *(ref)*		
Both dairy and beef	0.429	<0.001	0.301–0.611	0.454	<0.001	0.310–0.664
Dairy cattle only	0.739	0.352	0.391–1.397	0.601	0.189	0.281–1.285
Belonging to a cattle cooperative						
No	1 *(ref)*			1 *(ref)*		
Yes	1.865	<0.001	1.336– 2.603	2.049	<0.001	1.413–2.970
Listen to radio on cattle management						
No	1 *(ref)*			1 *(ref)*		
Yes	2.615	<0.001	1.845–3.706	2.279	<0.001	1.561–3.327

Hosmer-Lemeshow goodness of fit test statistic for attitude: 4.22, *p* = 0.8366; ROC curve – 0.7290.

As shown in Table 8, farmers who kept dairy cattle only had higher odds of appropriate practices compared with those keeping beef cattle only (AOR = 2.02, 95% CI: 1.07–3.80, *p* = 0.030). Years of farming experience remained significantly associated with practice levels. Farmers with 11–20 years of experience (AOR = 0.49, 95% CI: 0.31–0.79, *p* = 0.003) and those with more than 21 years of experience (AOR = 0.46, 95% CI: 0.28–0.74, *p* = 0.002) had lower odds of reporting appropriate practices compared with farmers with 0–5 years of experience.

**Table 8:** Logistic regression analyses of the independent factors associated with practices regarding AMU and AMR.

Predictor variables	Practice					
	Unadjusted			Adjusted		
	Odds ratio	*p*-value	Confidence interval	Odds ratio	*p*-value	Confidence interval
Type of cattle milked						
Beef cattle only	1 *(ref)*			1 *(ref)*		
Both dairy and beef	1.048	0.785	0.746–1.472	1.079	0.677	0.756–1.539
Dairy cattle only	2.442	0.004	1.335–4.468	2.017	0.030	1.072–3.797
Years of experience						
0–5 years	1 *(ref)*			1 *(ref)*		
6–10 years	0.595	0.030	0.372–0.952	0.639	0.065	0.399–1.028
11–20 years	0.453	0.001	0.287–0.714	0.493	0.003	0.310–0.786
>21 years	0.414	<0.001	0.258–0.665	0.456	0.002	0.280–0.741
Listen to radio on cattle management						
No	1 *(ref)*			1 *(ref)*		
Yes	1.291	0.149	0.912–1.827	1.384	0.083	0.958–2.000

Hosmer-Lemeshow goodness-of-fit test statistic for practice−5.13, *p* = 0.7439; ROC curve – 0.6096.

## 4 Discussion

This study provides important insights into the KAP related to antimicrobial use (AMU) and antimicrobial resistance (AMR) among smallholder dairy farmers in Zambia. The findings revealed limited knowledge of AMR, moderately positive attitudes, and suboptimal practices. Knowledge had a significant positive association with attitudes, whereby, those with good appropriate knowledge also had positive attitude, but its association with practices was not statistically significant. However, attitude had a significant positive association with practices. In this study, peer reliance for the treatment of cattle with antibiotics was prevalent. Farmers trusted other farmers as sources of both antimicrobials and advice, influencing the decision making around AMU. Further, withdrawal periods were adhered to in milk but not for meat consumption. Lastly, being part of a cooperative and listening to radio was associated with adequate knowledge and positive attitudes but not with appropriate practices.

In this study, a minority of farmers demonstrated appropriate knowledge of AMU and AMR, with the majority being unfamiliar with the concept of antibiotic resistance. These findings are consistent with studies in Ethiopia, where most farmers had poor knowledge on AMU, AMR and antibiotic residues (6). Levels lower than 60% were also reported in Kenya where only slightly over half of participants demonstrated correct knowledge of AMU/AMR (21). Similar misconceptions have also been reported by Dhangar et al. (29) who show that farmers believed that antibiotics were effective against all diseases. The low knowledge levels observed in this study may reflect the generally low levels of formal education among participants, as previous research has shown that limited schooling is often associated with misconceptions regarding AMU and AMR (30). Comparable patterns of low baseline knowledge have also been documented among secondary school students in Tanzania (31). The need for educating farmers on the use of antimicrobials in agriculture and the livestock industry in particular has been emphasized by the WHO African region (2017–2021).

Nevertheless, most farmers in this study expressed willingness to follow veterinary prescriptions and reported moderately positive attitudes consistent with findings from other settings (6, 29). Despite this, many still considered purchasing antibiotics without a prescription from agro-stores acceptable. Over the counter (OTC) access to antibiotics in Zambia has been widely reported even though antibiotics are legally prescription only drugs (32, 33) suggesting a gap between policy and practice. This discrepancy may reflect limited awareness regarding prescription requirements as well as inconsistent policy enforcement among retailers, as previous studies note (34). In this context, bulk purchasing and home storage of antimicrobials were commonly reported among dairy farmers (35). Similar behaviors, including prophylactic use of antibiotics, have also been documented among poultry farmers in Zambia (36). Although more than two-thirds (66.9%) of farmers in this study recognized AMR as a concern for cattle health, nearly half (48.8%) believed that antibiotic resistance had no implications for human health. This limited awareness of animal – human transmission risks is consistent with findings from other studies in Ethiopia (37).

Despite moderately positive attitudes, suboptimal AMU and AMR practice levels were reported. Farmers described sourcing antibiotics from peers, using antibiotics prophylactically, and discontinuing treatment before course completion. Similar practices have been reported in Rwanda, where 83.9% of farmers obtained antimicrobials from friends or neighbors (23), and in Zambia's poultry sector, where antibiotic use was nearly universal and mostly unsupervised (38, 39). In this study, most farmers reported adherence to milk withdrawal periods, contrasting with findings from Nyokabi et al. (40) where few farmers (6.7%) discarded milk from treated cows, often citing ‘greed' and economic considerations. However, adherence to withdrawal periods for meat consumption was substantially lower (24%). Previous studies have associated milk withdrawal compliance with factors such as milk rejection at collection centers, the length of withdrawal periods and perceived health effects of residues (7). Generally, compliance with withdrawal recommendations has been reported to be lower in settings with limited regulatory oversight (41). We also acknowledge that social desirability bias may have inflated the self-reported adherence to milk withdrawal in this study.

This study also found that belonging to a cooperative was associated with knowledge and attitude but not with practice. The reliance on fellow farmers for advice on treatment with antibiotics highlights both a risk and an opportunity. On one hand, peer networks may reinforce unsafe practices while on the other hand, they could serve as channels to promote stewardship practices (42). Studies have shown that farmer education delivered through cooperatives and community networks is significantly associated with improved preventive practices and reduced inappropriate AMU (6, 29). Similarly, listening to radio programs on cattle management was associated with appropriate knowledge and positive attitudes but not with practices. While radio has been associated with farmers' knowledge and attitudes, this may not always lead to desirable practices because knowledge translation into action may be constrained by several structural factors including the way radio programs are designed - lacking engagement and clear calls to action (43, 44). For instance, interactive and entertainment-based radio approaches such as drama series, have been reported to encourage reflection and behavior change among smallholder farmers when content is relatable and engaging (45).

Interestingly KAP level correlations were also noted, the significant correlation between knowledge and attitude supports behavioral theories that suggest that knowledge shapes perception, but external barriers influence behavior (46). In most sub-Saharan African countries, translating attitude into prudent antibiotic use practices is challenged by poor veterinary services leading farmers to rely on agrovets or experience for guidance (47). The lack of relationship between knowledge and practices observed in this study is consistent with other reports that show that correct knowledge did not reliably lead to desirable practices (6, 21). This persistent gap may reflect the presence of systematic drivers such as economic pressures, over-the-counter availability and rooted norms (17).

### 4.1 Limitations

We are aware that this study has some limitations. Due to the cross-sectional design of this study, causal relationships between socio-demographic factors and KAP levels of smallholder dairy farmers cannot be established. The associations observed should be interpreted cautiously, as temporal relationships between exposure and outcome could not be determined. Biases such as recall bias were possible and it was dealt with by asking questions related to the current knowledge, attitudes and practices of the dairy farmers within their settings. Responses were also prone to social desirability, which was minimized by creating rapport with respondents and reassuring them that their responses would not affect their daily operations. Since the study used structured questions, it could not probe underlying social cultural drivers of knowledge, attitudes and practices such as the social norms. The relatively small proportion of dairy-only farmers may have influenced the precision of estimated associations; therefore, these findings should be interpreted with appropriate caution. Finally, the classification of knowledge, attitudes, and practices into categorical levels was based on predefined percentage thresholds. Although these thresholds were adopted from comparable studies to enhance interpretability and comparability, the findings in this study should be interpreted with caution.

## 5 Conclusion

The study highlighted low levels of KAP related to AMU and AMR, levels that may reflect antimicrobial misuse that may lead to the development and spread of AMR. Considering that knowledge was not associated with practices, the study suggests that antimicrobial stewardship programs that incorporate the promotion of positive attitudes and create enabling farm level environments could support and sustain prudent antimicrobial use. Using local farmers, cooperatives and radio as avenues for extension services in Zambia could potentially improve KAP regarding AMU and AMR, studies to show this hypothesis are recommended. We also recommend that further research should be conducted to understand the drivers of antimicrobial consumption, milk antimicrobial residue and antimicrobial resistance patterns among smallholder dairy farming systems.

## Data Availability

The raw data supporting the conclusions of this article will be made available by the authors, without undue reservation.
